# Why We May Need Higher Doses of Beta-Lactam Antibiotics: Introducing the ‘Maximum Tolerable Dose’

**DOI:** 10.3390/antibiotics11070889

**Published:** 2022-07-04

**Authors:** Sofie A. M. Dhaese, Eric A. Hoste, Jan J. De Waele

**Affiliations:** 1 Department of Intensive Care Medicine, Ghent University Hospital, 9000 Ghent, Belgium; 2 Department of Internal Medicine and Pediatrics, Ghent University Hospital, 9000 Ghent, Belgium

**Keywords:** beta-lactam antibiotics, pharmacokinetics, pharmacodynamics, ICU, critically ill

## Abstract

The surge in antimicrobial resistance and the limited availability of new antimicrobial drugs has fueled the interest in optimizing antibiotic dosing. An ideal dosing regimen leads to maximal bacterial cell kill, whilst minimizing the risk of toxicity or antimicrobial resistance. For beta-lactam antibiotics specifically, PK/PD-based considerations have led to the widespread adoption of prolonged infusion. The rationale behind prolonged infusion is increasing the percentage of time the beta-lactam antibiotic concentration remains above the minimal inhibitory concentration (%*f*T_>MIC_). The ultimate goal of prolonged infusion of beta-lactam antibiotics is to improve the outcome of infectious diseases. However, merely increasing target attainment (or the %*f*T_>MIC_) is unlikely to lead to improved clinical outcome for several reasons. First, the PK/PD index and target are dynamic entities. Changing the PK (as is the case if prolonged instead of intermittent infusion is used) will result in different PK/PD targets and even PK/PD indices necessary to obtain the same level of bacterial cell kill. Second, the minimal inhibitory concentration is not a good denominator to describe either the emergence of resistance or toxicity. Therefore, we believe a different approach to antibiotic dosing is necessary. In this perspective, we introduce the concept of the maximum tolerable dose (MTD). This MTD is the highest dose of an antimicrobial drug deemed safe for the patient. The goal of the MTD is to maximize bacterial cell kill and minimize the risk of antimicrobial resistance and toxicity. Unfortunately, data about what beta-lactam antibiotic levels are associated with toxicity and how beta-lactam antibiotic toxicity should be measured are limited. This perspective is, therefore, a plea to invest in research aimed at deciphering the dose–response relationship between beta-lactam antibiotic drug concentrations and toxicity. In this regard, we provide a theoretical approach of how increasing uremic toxin concentrations could be used as a quantifiable marker of beta-lactam antibiotic toxicity.

## 1. Introduction

Increasing drug resistance rates and the scarcity of new antibacterial drugs pose a serious threat for the clinical utility of antimicrobial drugs [1]. In response, Antimicrobial Stewardship Programs (ASP) were introduced to help preserve our antimicrobial armamentarium by interventions designed to ensure the appropriate use of antimicrobial drugs [2,3]. One of these interventions is dose-optimization, i.e., informed decision making regarding the optimal dose and dosing regimen for the individual patient [4].

The scientific advances in the field of antimicrobial dose optimization have mainly been determined by pharmacokinetic (PK) and pharmacodynamic (PD) principles. PK/PD is the science relating the effect of drug exposure (PK) to an outcome measurement (PD) [5]. For antibiotics specifically, PK/PD describes the drug exposure necessary to achieve bacterial cell kill, while limiting its side effects i.e., toxicity and antimicrobial resistance. Beta-lactam antibiotics, amongst the most commonly prescribed antimicrobial drugs in the ICU, are a present-day example of how PK/PD considerations led to the adoption of alternative modes of infusion to optimize their use [6].

In recent years, a wealth of evidence emerged, demonstrating that the PK of beta-lactam antibiotics in critically ill patients is significantly different from the beta-lactam PK observed in healthy volunteers or non-critically ill patients [7]. The patients with sepsis and septic shock may have an increased or decreased drug clearance and an increased volume of distribution. Because of their hydrophilic nature and predominantly renal elimination, changes in kidney function and the volume of distribution profoundly impact the beta-lactam antibiotic PK [7,8]. As a result, several reports have illustrated subtherapeutic antibiotic drug concentrations in critically ill patients treated with standard dosing beta-lactam antibiotic drugs [9,10].

## 2. How PK/PD Is Currently Used to Optimize Dosing of Beta-Lactam Antibiotics in the Critically Ill

Beta-lactam antibiotics are considered time-dependent antibiotics and the time (T) that the unbound fraction (*f*) of the antibiotic drug remains above the minimal inhibitory concentration (MIC) is the PK/PD index of choice (*f*T_>MIC_) [11,12]. By convention, the magnitude of the PK/PD index necessary to achieve a certain outcome (for example a 3-log_10_ reduction of colony-forming units (CFU/mL)) is called the PK/PD target [5]. Importantly, the MIC is a value determined in the laboratory under highly standardized conditions that are very different from in vivo conditions; and the MIC therefore does not represent a concentration that can be compared with an in vivo drug concentration [13].

**Figure 1:**
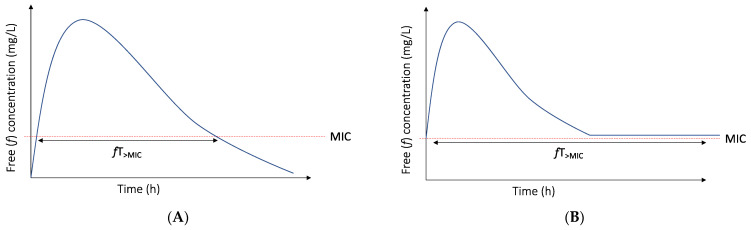
Time above the MIC for intermittent (**A**) and continuous (**B**) infusion with initial bolus.

The rationale for prolonged (i.e., both extended and continuous) infusion of beta-lactam antibiotics is extending the duration of infusion in order to increase the %*f*T_>MIC_ and target attainment rates (Figure 1). The ultimate goal of prolonged infusion is improving the outcome of the infection.

The ability of prolonged infusion to increase the %*f*T_>MIC_ has been clearly demonstrated [14,15]. Unfortunately, the benefit of prolonged infusion in terms of reduced mortality is still a matter of debate. Indeed, many clinical studies have evaluated intermittent versus prolonged infusion of beta-lactam antibiotics, but very few have evaluated mortality as an outcome parameter. Only two randomized clinical trials (RCTs) have demonstrated a lower mortality rate with a prolonged versus intermittent infusion of beta-lactam antibiotics in critically ill patients [16,17]. Other RCTs have demonstrated improved clinical cure rates [14,18], lower costs [19,20], a faster reduction of the APACHE (Acute Physiology and Chronic Health Evaluation) II score [21], increased microbiological success rates [22] or improved target attainment rates [15,23] with prolonged infusion, albeit without an effect on mortality. Two systematic reviews and one individual patient meta-analysis demonstrated lower mortality rates with prolonged as opposed to intermittent infusion in patients with sepsis and severe sepsis [24,25,26]. Currently, BLING III, a large multicenter trial comparing the 90-day all-cause mortality between intermittent and continuous infusion piperacillin and meropenem has almost finished recruitment, and the results are eagerly awaited [27].

## 3. Why We Need to Rethink the Use of Prolonged Infusion of Beta-Lactam Antibiotics to Improve the Outcome of Infection

### 3.1. The PK/PD Index and Target of Choice for Beta-Lactam Antibiotics Are Not Static Entities

**Figure 2:**
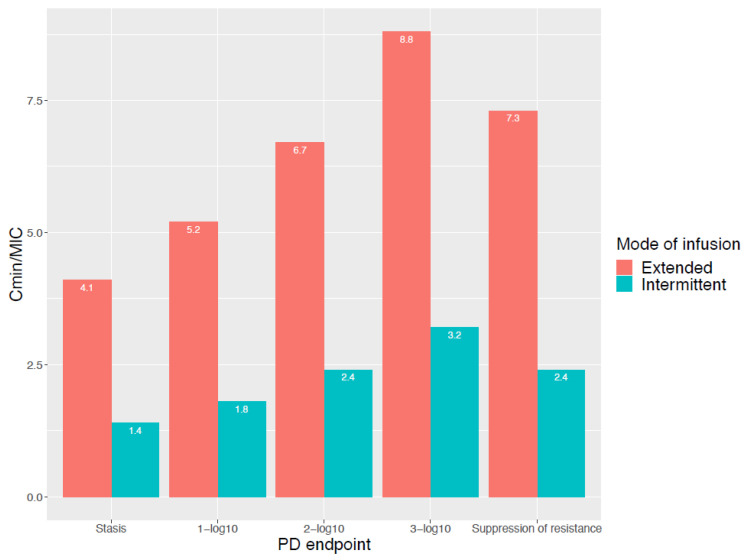
C_min_/MIC ratio for different PD endpoints and for both intermittent and extended infusion. Reproduced from Felton et al. [29].

Prolonging the duration of infusion to increase the target attainment depends on the assumption that the PK/PD index and target by itself are static and are independent of the mode of infusion used. However, this theory has been challenged, and attaining the same PK/PD target with a different mode of infusion does not necessarily imply an equal level of bacterial cell kill [28]. For example, Felton et al. [29] published an in vitro *Pseudomonas aeruginosa* hollow-fiber infection model for piperacillin. A dosing of 3, 9 and 17 g of piperacillin, either via intermittent (0.5 h infusion duration) or extended infusion (4 h infusion duration) was simulated. The targets (in C_min_/MIC ratios) reported for stasis, 1-, 2- and 3-log_10_ kill and the suppression of resistance for extended infusion were consistently higher compared with the targets documented for intermittent infusion (Figure 2). In addition, Sumi et al. [30] evaluated intermittent, extended and continuous infusion piperacillin/tazobactam in an in vitro dynamic hollow-fiber infection model against ceftriaxone-resistant *Klebsiella pneumoniae*. For the Kp69 strain (with an MIC of 1 mg/L), a C_min_/MIC ratio of 1.09 with intermittent infusion was sufficient to avoid resistance development, while for the extended infusion a C_min_/MIC ratio of 3.18 was necessary. These examples illustrate that different PK/PD targets may apply for the same reduction of CFU when different modes of infusion are used.

The concept of dynamic PK/PD indices and targets in terms of changing beta-lactam antibiotic concentrations have previously been described [31,32,33,34], and the idea of a dynamic PK/PD relationship, linking changing antibiotic concentrations to bacterial kill or growth over time, is well established [5,13,33,35,36]. However, we do not generally consider that, for a different mode of infusion of the same antibiotic, different indices and targets may apply. When comparing the probability of target attainment between intermittent and continuous infusion, which implies a fundamentally different concentration–time curve, it is usually assumed that the PK/PD index and target remain the same [6,28,35,36,37].

Intriguingly, the optimal PK/PD index is also dependent on PK, as described by Nielsen et al. [38] and Kristofferson et al. [33]. These authors argue that the choice of *f*T_>MIC_ as the PK/PD index of beta-lactam antibiotics is related to the short half-life (and therefore the PK) of most of these drugs. In situations where the half-life is prolonged, for example in patients with kidney failure, *f*AUC/MIC was found as the best predictor of the antibacterial effect of beta-lactam antibiotics [33]. Even more so, when other drugs (from different antibiotic classes, such as fluoroquinolones or glycopeptides) were used for simulation, with a half-life equal to the half-life of benzylpenicillin, *f*T_>MIC_ was the PK/PD index best related to the antibacterial efficacy [38]. The fact that the PK/PD index is a summary endpoint, dependent on both PK and PD, has also been demonstrated for drugs other than beta-lactam antibiotics. For example, in a lung and thigh infection neutropenic mouse model of Craig et al. [39], *f*T_>MIC_ is the PK/PD index of choice for amikacin in mice with a normal kidney function (half-life of 18.5–32.5 min), as opposed to *f*AUC/MIC in mice with an impaired kidney function (half-life of 93.3–121 min).

### 3.2. Bacterial Cell Kill Is Not the Only Goal

An optimal dosing regimen would allow maximal antibacterial effect, whilst minimizing drug toxicity and the risk of resistance development. Nevertheless, most of our beta-lactam antibiotic dosing regimens were based upon PK/PD targets and indices for bacterial cell kill alone. However, the recent literature has illustrated that we may need different antibiotic exposures (illustrated by different PK/PD targets and indices) for the suppression of resistance, as opposed to bacterial cell kill [40,41]. Moreover, several authors have advocated for the mutant prevention concentration (MPC) instead of the MIC as the PD endpoint for the suppression of resistance [42]. The MPC is the concentration that prevents the growth of first-step resistant mutants. This concept is based on the idea that a large initial bacterial burden has a high probability of harboring a first-step mutant. The mutant selection window (MSW) is defined as a range of concentrations between the MIC and the MPC. The concentrations within the MSW are expected to promote the selection of resistance [43,44]. However, the MIC may not necessarily be correlated to the MPC or MSW, and using MIC as a PD denominator to describe the suppression of resistance might therefore not be appropriate [45]. If the MIC is not a good PD denominator to describe the risk for resistance development, increasing the %*f*T_>MIC_ with a prolonged infusion of beta-lactam antibiotics will be of no use when resistance development is concerned. Indeed, determinants other than the mode of infusion, such as the pathogen involved, the duration of therapy and the initial inoculum size, may be much more important for regrowth [41].

Finally, a PK/PD index or target linked to bacterial cell kill will tell us nothing about the risk of toxicity, as toxicity for a patient is not associated with susceptibility. Hence, using a PK/PD target (for example C_ss_ 10 times the MIC) to avoid toxicity is not relevant. As Lau et al. [46] and others [47,48,49,50,51] observed, beta-lactam drug toxicity is most likely linked to the through concentrations. This finding is especially worrisome, as prolonged infusions of beta-lactam antibiotics will, by definition, lead to higher through (or, in the case of continuous infusion, steady state) concentrations.

## 4. Introducing the ‘Maximum Tolerable Dose’ to Overcome the above Limitations

Based on the above considerations, and from a purely clinical point of view, using a ‘maximum tolerable dose’ could be an attractive alternative for beta-lactam dosing. It would maximize the cell kill, avoid resistance development and alleviate the need for complex dosing regimens in response to dynamic PK/PD indices and targets (of which most were derived from preclinical experiments). In addition, higher dosing will lead to higher tissue concentrations, which is important in critically ill patients, given the high variability of tissue penetration to different foci of infection [7,52]. Finally, using the MTD may also facilitate shortening the duration of the antimicrobial therapy.

Translation into practice would require knowledge of the concentrations associated with beta-lactam toxicity and, preferably, toxicity would be easily measurable [5]. To date, there is very little information available regarding beta-lactam antibiotic toxicity and dose–response relationships. Known beta-lactam adverse reactions are hypersensitivity, nephrotoxicity, myelotoxicity, neurotoxicity, hepatotoxicity and *Clostridioides difficile* infection [53]. Of these adverse reactions, the evidence for an exposure–response relationship is strongest for neurotoxicity. Several beta-lactam antibiotic concentrations have been linked to neurotoxicity (Table 1), although the beta-lactam antibiotic subclass prescribed is also an important predictor. For example, the proconvulsive effect of cefepime is estimated to be ten to fifteen times as high when compared with meropenem and piperacillin respectively [54]. Approximately 10–15% of the ICU patients receiving beta-lactam antibiotic drugs develop neurotoxicity, but this usually soon resolves after discontinuation or dose reduction [53,54]. The problem with beta-lactam antibiotic neurotoxicity, especially in critically ill patients, is the fact that it is difficult to distinguish from other causes of neurologic changes, such as brain damage, encephalopathy, sepsis, other toxic medications, delirium, etc. Unfortunately, no neurologic symptom is specific for beta-lactam-induced neurotoxicity [54].

Crystal nephropathy, which is a result of antimicrobial precipitation and crystallization in the renal tubuli has been documented with high amoxicillin concentrations, but is assumed to be very rare and a specific drug level linked to crystallization has not been defined [55,56].

Hypersensitivity is a common side-effect of beta-lactam antibiotics, but is likely not linked to the dosing regimen or drug concentration. Acute interstitial nephritis and drug-induced liver injury (DILI) are immune-mediated idiosyncratic reactions, and it is therefore assumed that these reactions are also not linked to the drug concentration. Whether or not myelotoxicity is dose-dependent is a matter of debate [57].

**Table 1:** Beta-lactam neurotoxicity levels.

Beta-Lactam Antibiotic	Neurotoxicity Levels Reported	References
Cefepime	20 mg/dL (II, t), 21.6 mg/dL (II, t), 22 mg/dL (II, t), 36 mg/dL (II, t), 63.2 mg/dL (CI, ss)	[49,50,51,58,59]
Piperacillin/tazobactam	361.4 mg/dL (II, t),157 mg/dL (CI, ss)	[47,60]
Meropenem	64.2 mg/dL (II, t)	[47]
Flucloxacillin	125.1 mg/dL (II, t)	[47]

II: intermittent infusion; CI: continuous infusion; t: trough concentration; ss: steady state concentration.

## 5. What Other Options Might We Have to Assess Beta-Lactam Antibiotic Toxicity?

Not unsurprisingly, beta-lactam through concentrations are related to a decline in kidney function [47,50,51]. Indeed, beta-lactam antibiotics are predominantly renally eliminated, and reduced elimination will lead to higher serum levels [8]. However, other aspects of a decline in kidney function, such as uremic toxin accumulation, might also be relevant with regards to beta-lactam toxicity. Uremic toxins are endogenous waste products that are secreted by the kidney in healthy individuals. In patients with kidney disease, uremic toxins accumulate, leading to symptoms of uremia, such as anorexia, lethargy and altered mental function [61]. Uremic toxins are divided into small, water-soluble toxins, middle molecules and protein-bound uremic toxins (PBUTs) [62]. The clearance of PBUTs is more dependent on tubular secretion than glomerular filtration [63]. The tubular secretion of these toxins is mediated by basolateral and luminal transporters expressed on the tubular epithelial cells. More specifically, the organic anion transporter 1 (OAT1) and the organic anion transporter 3 (OAT3) are the main transporters responsible for the basolateral uptake of PBUTs from renal blood. For several β-lactam antibiotics, renal elimination is assumed to consist of both glomerular filtration, as well as tubular secretion via the basolateral OAT1 and OAT3 transporters [64,65,66,67,68,69]. It is, for example, assumed that as much as 50 to 75% of the renal elimination of piperacillin, a broad spectrum β-lactam antibiotic, is governed by tubular secretion [70]. Unlike glomerular filtration, tubular secretion is a competitive process with the potential for interactions between several drugs and/or endogenous solutes, in this case, an interaction between PBUTS and beta-lactam antibiotic concentrations [71].

With respect to the theory of the ‘maximum tolerable dose’, modeling beta-lactam concentrations (PK) as well as modeling uremic toxin concentrations (PD) as two dynamic parameters (pharmacokinetic/toxicodynamic modeling), analogous to the PK/PD models incorporating dynamic bacterial growth in response to changing antibiotic concentrations, may circumvent the issues we currently experience with static PK/PD indices and targets [5].

## 6. Conclusions

An ideal antibiotic dosing regimen maximizes bacterial cell kill, whilst minimizing drug toxicity and the risk for resistance development. In critically ill patients, the finding of low beta-lactam antibiotic concentrations due to PK variability has led to the adoption of prolonged infusion to increase target attainment. From a purely PK/PD point of view, increasing the duration of the infusion to increase the %*f*T_>MIC_ will not, by definition, lead to increased bacterial cell kill given that the PK/PD index and target are not static entities. Moreover, merely prolonging the duration of infusion in an attempt to increase the %*f*T_>MIC_ is likely irrelevant when it comes to suppression of regrowth and avoidance of toxicity. In the future, administering a maximum tolerable dose instead of a (minimum) dose that has been developed to achieve a predefined PK/PD target for efficacy only, may help preserve our antimicrobial armamentarium. Currently, the specific levels of beta-lactam drug toxicity are ill-defined and therefore research focusing on the pharmacodynamics of beta-lactam antibiotic toxicity is urgently needed. A first step in this process should be measuring the uremic toxin concentrations in patients receiving beta-lactam antibiotics. These data can then be used to develop a pharmacokinetic/toxicodynamic model, which in turn could inform clinicians on the maximum tolerable dose. The patients with advanced kidney disease are at risk of both high uremic toxin concentrations, as well as high beta-lactam antibiotic concentrations and therefore represent a study population of interest for the purpose of developing such a pharmacokinetic/toxicodynamic model.

## Data Availability

Not applicable.
